# Stable, reproducible, and binder-free gold/copper core–shell nanostructures for high-sensitive non-enzymatic glucose detection

**DOI:** 10.1038/s41598-022-23504-2

**Published:** 2022-11-08

**Authors:** Hossein Siampour, Sara Abbasian, Ahmad Moshaii, Amir R. Amirsoleimani

**Affiliations:** 1 Department of Physics, Tarbiat Modares University, P.O Box 14115-175, Tehran, Iran; 2 Department of Sensor and Biosensor, Faculty of Interdisciplinary Sciences and Technologies, Tarbiat Modares University, P.O. Box 14115-336, Tehran, Iran

**Keywords:** Sensors and biosensors, Synthesis and processing

## Abstract

The core–shell non-enzymatic glucose sensors are generally fabricated by chemical synthesis approaches followed by a binder-based immobilization process. Here, we have introduced a new approach to directly synthesis the core–shell of Au@Cu and its Au@Cu_x_O oxides on an FTO electrode for non-enzymatic glucose detection. Physical vapor deposition of Au thin film followed by thermal annealing has been used to fabricate Au nanocores on the electrode. The Cu shells have been deposited selectively on the Au cores using an electrodeposition method. Additionally, Au@Cu_2_O and Au@CuO have been synthesized via post thermal annealing of the Au@Cu electrode. This binder-free and selective-growing approach has the merit of high electrooxidation activity owing to improving electron transfer ability and providing more active sites on the surface. Electrochemical measurements indicate the superior activity of the Au@Cu_2_O electrode for glucose oxidation. The high sensitivity of 1601 μAcm^−2^ mM^−1^ and a low detection limit of 0.6 μM are achieved for the superior electrode. Additionally, the sensor indicates remarkable reproducibility and supplies accurate results for glucose detection in human serums. Moreover, this synthesis approach can be used for fast, highly controllable and precise fabrication of many core–shell structures by adjusting the electrochemical deposition and thermal treatment parameters.

## Introduction

Reliable, rapid and cost-effective blood glucose sensing is a significant challenge owing to worldwide growing diabetes mellitus^1,2^. The sensitive glucose detection is also important for food, drug and environmental monitoring^3–5^. Noticeable efforts have been dedicated to developing enzymatic glucose sensors with high sensitivity and selectivity^6–9^. However, there are some limitations to overcome, including high cost, complicated enzyme immobilization procedure, and low stability of the enzymes^10–12^.

Currently, highly sensitive non-enzymatic glucose sensors with fast response time, low cost and high stability have attracted great attentions^13–15^. Owing to high surface area and enhanced electrocatalytic properties, various metal and metal oxide nanostructures have been considerably used in the fabrication of non-enzymatic glucose sensors^16–19^. However, some limitations of mono-material constructed electrodes obstruct their practical application in glucose sensors. For example, noble metals such as Ag, Pt and Au exhibited excellent catalytic activity but suffered from some disadvantages, including high cost, surface poisoning, and low selectivity^20,21^. On the other hand, transition metal oxides like Cu_2_O, CuO, NiO and Co_2_O_3_ are more interested due to their low cost, abundancy and high stability^22–25^. However, their poor electrical conductivity and charge transfer ability are challenging issues in their applications^21,26^. Accordingly, using metal oxides modified with metal nanoparticles^27–30^ and metal/metal oxide hybrid core–shell structures^18,31–33^ seem to provide a significant improvement in glucose sensing performance. Among various nanostructures, Au@copper oxide core-shells are a promising candidate for glucose detection since excellent charge transfer ability of Au and superior electrocatalytic activity of copper oxides integrate to novel synergistic properties^34,35^.

Synthesis of core shell structures is generally carried out by the solution-phase methods followed by their immobilization on a supporting electrode. It includes reducing metal ions in the presence of core seeds and shape-directing additives. This approach suffers from hindering electrocatalytic activity due to existence of surfactant and binding additives. Accordingly, other fabrication methods especially those based on the direct deposition of core–shell structures on a conductive substrate are more interested^36–38^.

Here, a new approach has been raised to directly fabricate the core–shell structures on a conductive electrode. Core–shell of Au@Cu and Au@Cu_x_O have been constructed directly on an FTO electrode by incorporating physical vapor deposition, electrochemical deposition and thermal treatments. The results show selective deposition of Cu shell on the Au nanocores without any accumulation. The proposed direct and binder-free approach boosts the electron transfer ability and reaction capacity of the shell. Selective formation of core–shell without accumulation improves electrooxidation activity of the sensor due to providing more active site and facilitating glucose diffusion. In addition, high repeatability and controllability of fabrication processes are achieved. The results indicate the superiority of glucose oxidation activity of Au@Cu_2_O in comparison to Au@Cu and Au@CuO. Finally, a high sensitivity of 1601 μAcm^−2^ mM^−1^ and a low detection limit of 0.6 μM are obtained for Au@Cu_2_O.

## Results

1. Characterization of Au nanocores

**Figure 1:**
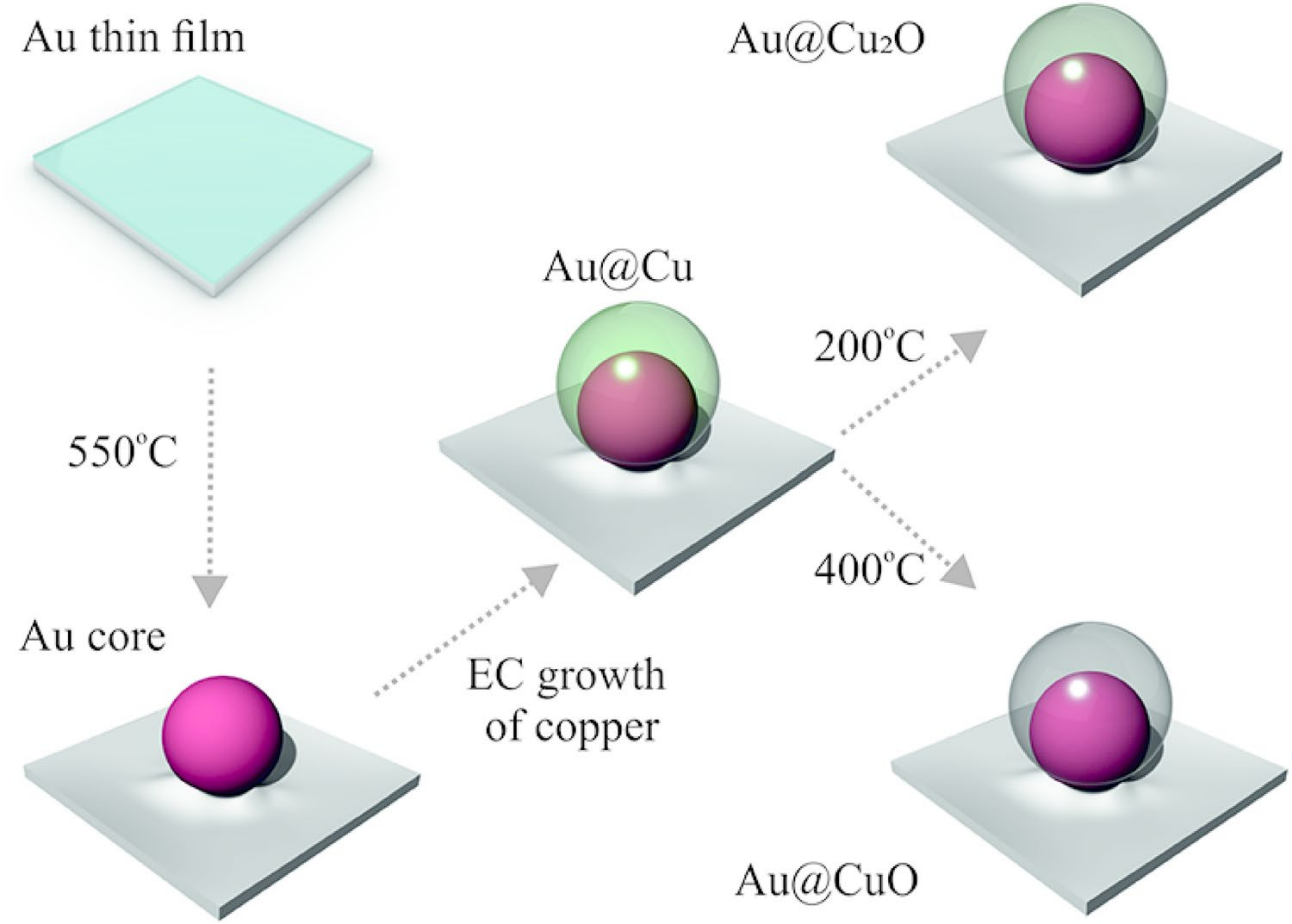
Schematic illustration of the fabrication procedure of Au@Cu and Au@Cu_x_O including physical vapor deposition of Au thin film, fabrication of the Au nanocore by thermal annealing, the electrochemical deposition of copper on the Au nanocore and the final thermal treatment.

The fabrication approach of Au@Cu and Au@Cu_x_O directly on the FTO substrate is illustrated schematically in Fig. 1. Initially, Au nanocores were fabricated via physical vapor deposition of different Au thin films followed by thermal annealing at 550 °C. To produce Au@Cu, a copper shell was electrochemically deposited on the Au core supported FTO substrate. The electrochemical deposition of the copper shell was performed under potentiostatic mode in 1 mM CuSO_4_ solution at room temperature. Eventually, to develop Au@Cu_2_O and Au@CuO, the as-prepared Au@Cu were annealed under the air atmosphere at 200 °C and 400 °C, respectively.

**Figure 2:**
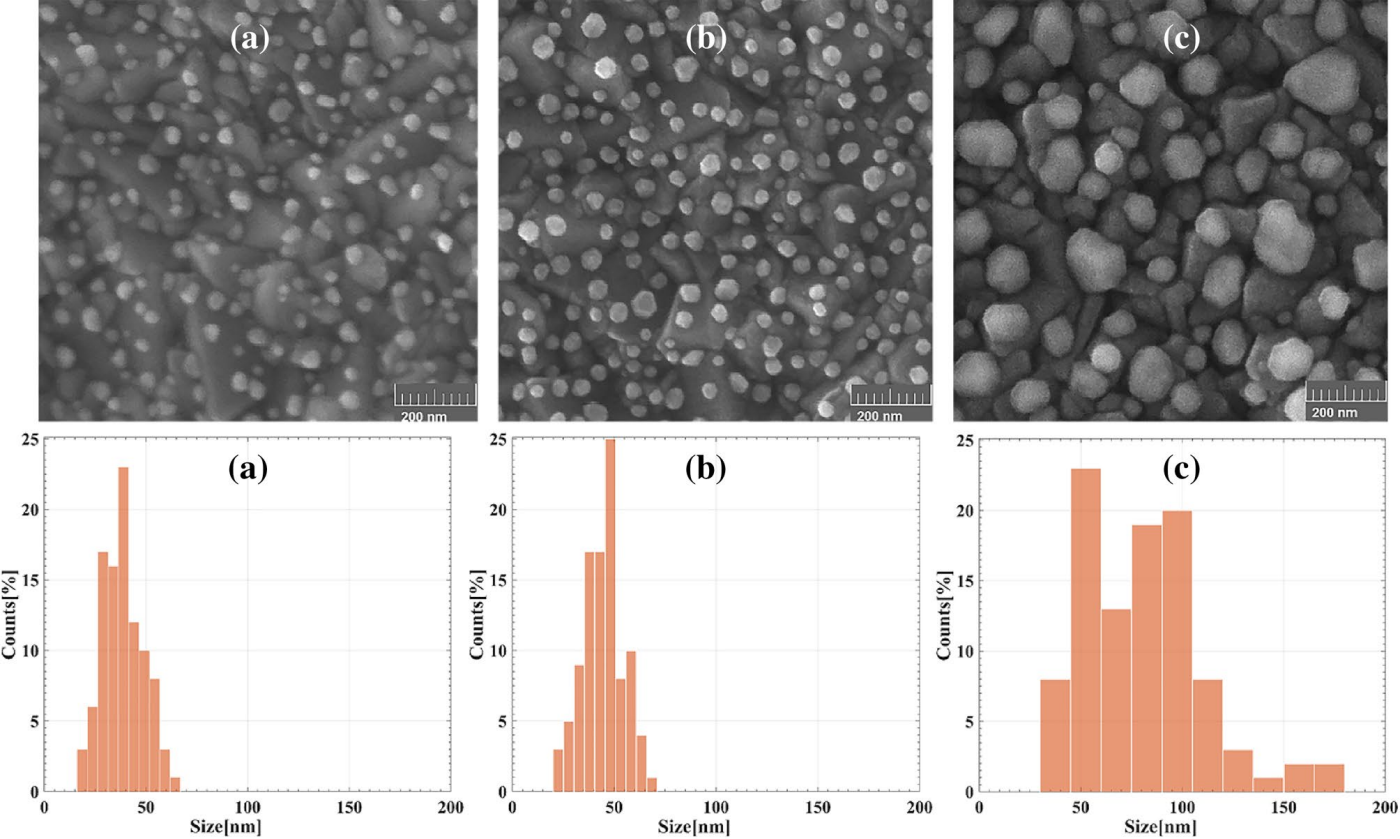
SEM images and size distribution histograms of Au nanocores fabricated by thermal annealing of Au thin films with different deposition thicknesses of (**a**) 2.5 nm, (**b**) 5 nm and (**c**) 10 nm.

Figure 2 shows SEM images and size distribution histograms of the Au cores with various film thicknesses. A random array of well-separated Au nanocores are fabricated on the FTO substrate for all deposition thicknesses of 2.5, 5 and 10 nm. As indicated in Fig. 2, by the increment of the deposition thickness, the diameter of nanocores increases. For the deposition thicknesses of 2.5, 5 and 10 nm, the average diameters of Au nanocore are 38, 44 and 80 nm, respectively.

To obtain the optimum Au deposition thickness for core formation, the electrochemical behaviors of the Au nanocores with various thicknesses have been measured by cyclic voltammetry (CV) and electrochemical impedance spectroscopy (EIS). The CV curves have been measured at the potential range from − 0.2 to 0.6 V in the solution (0.1 M KCl) containing redox couple of [Fe (CN)_6_]^−3/−4^ (2.5 $\times$ 10^−3^ M), at the scan rate of 50 mVs^−1^. The EIS measurements have been carried out at the frequency range of 100 kHz to 0.1 Hz with 5.0 mV AC perturbation.

The CVs curves of the bare FTO and different core supported FTO electrodes are shown in Fig. S1. A pair of redox peaks is observed for the bare FTO with the anodic (E_pa_) and cathodic (E_pc_) current peak potentials of 0.38 V and 0.06 V, respectively. The peak-to-peak separation potential (ΔE_p_ = E_pa_ − E_pc_) is obtained as 0.33 V for the bare FTO. Modification of the FTO electrode by Au nanocores leads to incrementing the intensity of current peaks and reducing ΔE_p_ for all deposition thicknesses. The ΔE_p_ is obtained as 0.23, 0.19 and 0.21 V for deposition thicknesses of 2.5, 5 and 10 nm, respectively. As is observed in Fig. S1a, the Au nanocores with 5 nm thickness exhibit the highest current intensity and lowest ΔE_p_.

Figure S1b of the electronic supplementary information (ESI) shows the Nyquist plots (EIS curves) of the bare FTO and the core supported FTO electrodes with various deposition thicknesses. For the bare FTO, a large electron-transfer resistance (identified as Ret) of 2.4 KΩ is observed indicating high resistance of the bare FTO surface against charge transfer. Modification of FTO electrodes by Au nanocores with 2.5 nm deposition thickness results in a notable decrease in Ret (0.8 KΩ). A further decrease in Ret (0.24 KΩ) is observed for Au nanocores with 5 nm deposition thicknesses indicating increment of the active surface area of the electrode. For the deposition thickness of 10 nm, the Ret of 0.4 KΩ is obtained. Considering the results of Fig. S1, the Au nanocores with 5 nm deposition thickness show the highest charge transfer performance and were selected for copper shell deposition in the following experiments.

2. Characterization of Au@Cu and Au@CuxO structures

**Figure 3:**
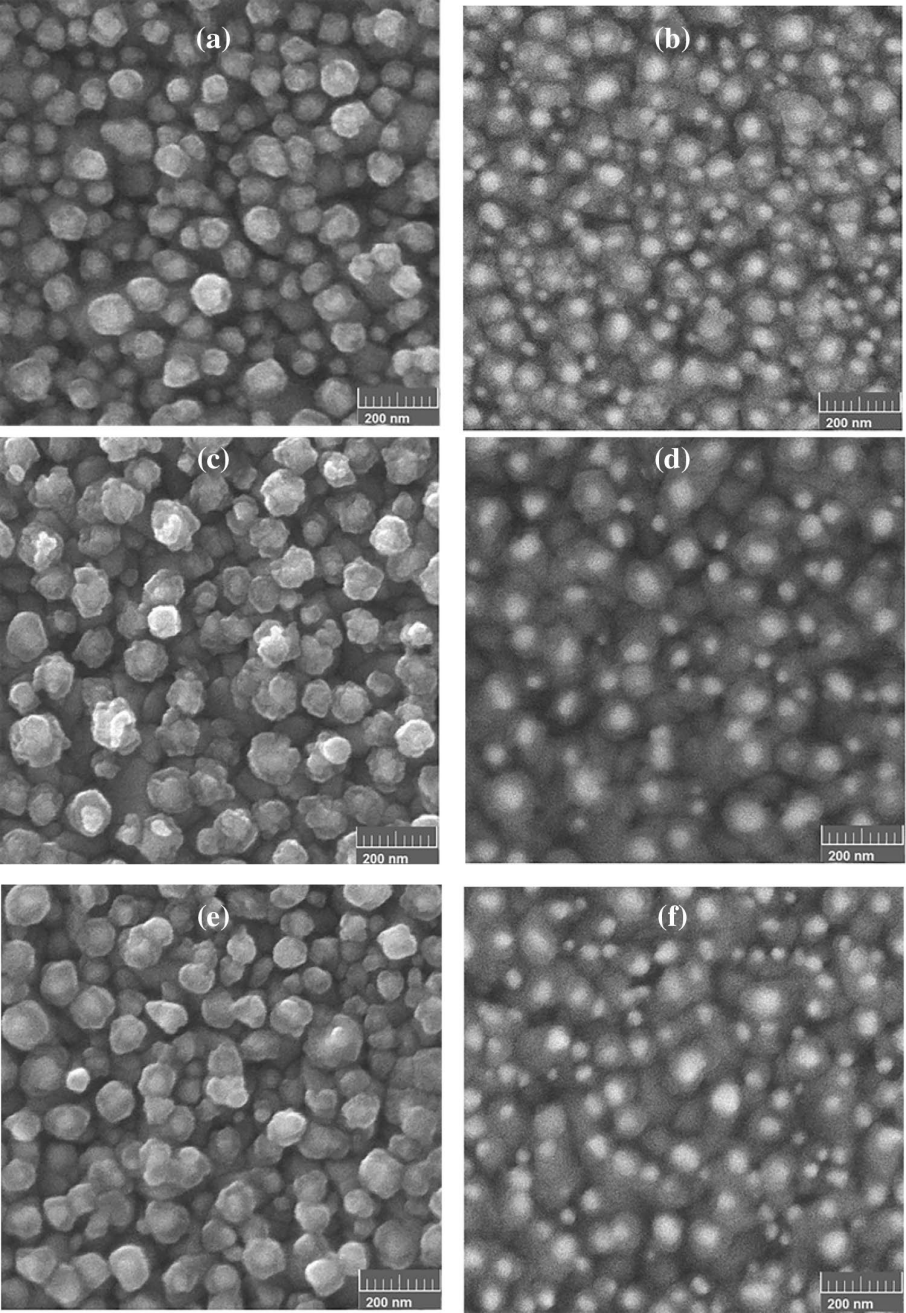
SEM and BSE images of (**a**,**b**) the copper deposited on the Au nanocore at the potential − 0.45 V for 600 s in 1 mM CuSO4 electrolyte solution, (**c**,**d**) the as-prepared Au@Cu at the temperature of 200 °C and (**e**,**f**) the as-prepared Au@Cu at the temperature of 400 °C.

Figures S2 and 3 show SEM images of the copper electrochemically deposited on the bare and the Au nanocore electrodes before and after thermal annealing processes. As shown in Figs. S2 and 3a, the growth pattern, shape and surface coverage of the deposited copper considerably differ for the bare and the Au core electrodes. For bare FTO, sparse cubic Cu nanostructures with a mean size of about 200 nm are formed on the electrode. In the case of the Au core electrodes, considering the contrast between the inner Au cores and the outer shell confirms the formation of the Cu shell on the Au nanocores. To get a better insight, backscattering electron (BSE) image of Au@Cu has been recorded, as shown in Fig. 3b. All the Au cores are encapsulated in uniform Cu shells. In fact, the epitaxial nucleation of Cu on the Au surface occurs due to epitaxial growth of Au core and Cu shell^39–41^. In addition, the higher surface energy of the Au nanocores compared to the FTO defect sites leads to the competitive deposition of Cu on the Au surface^42,43^. Therefore, the proposed method results in the direct and binder free formation of a dense array of Au@Cu structure with the size distribution range of 20–60 nm.

Thermal treatment of Au@Cu was carried out at the temperature of 200 °C and 400 °C to construct Au@Cu_2_O and Au@CuO, respectively. Figure 3c–f shows SEM and BSE images of thermally treated Au@Cu. Thermal treatment in the air atmosphere induces changes in the shell structures owing to the oxidation of the Cu shell^44,45^. This causes that the sizes of Au@Cu_2_O and Au@CuO increase relative to the original Au@Cu.

To verify the selective deposition of Cu shell on Au nanocores, the chronoamperometry measurement was utilized since the details of the transient current can be obtained by this method^43,46^. Figure S3 illustrates the transient current–time curves during deposition of Cu on the bare and the core supported FTO electrodes. In the case of the bare FTO, both nucleation and growth processes are observable. In the early stages of deposition, the current increases due to the formation of the Cu nucleus on the bare FTO. For later times, the current decreases due to the FTO surface coverage by Cu nanostructures resulting in appearance of a peak in the current–time curve. For the core supported FTO electrode, the absence of the current peak relating to the nucleation process confirms direct and selective deposition of Cu on Au cores^43^.

**Figure 4:**
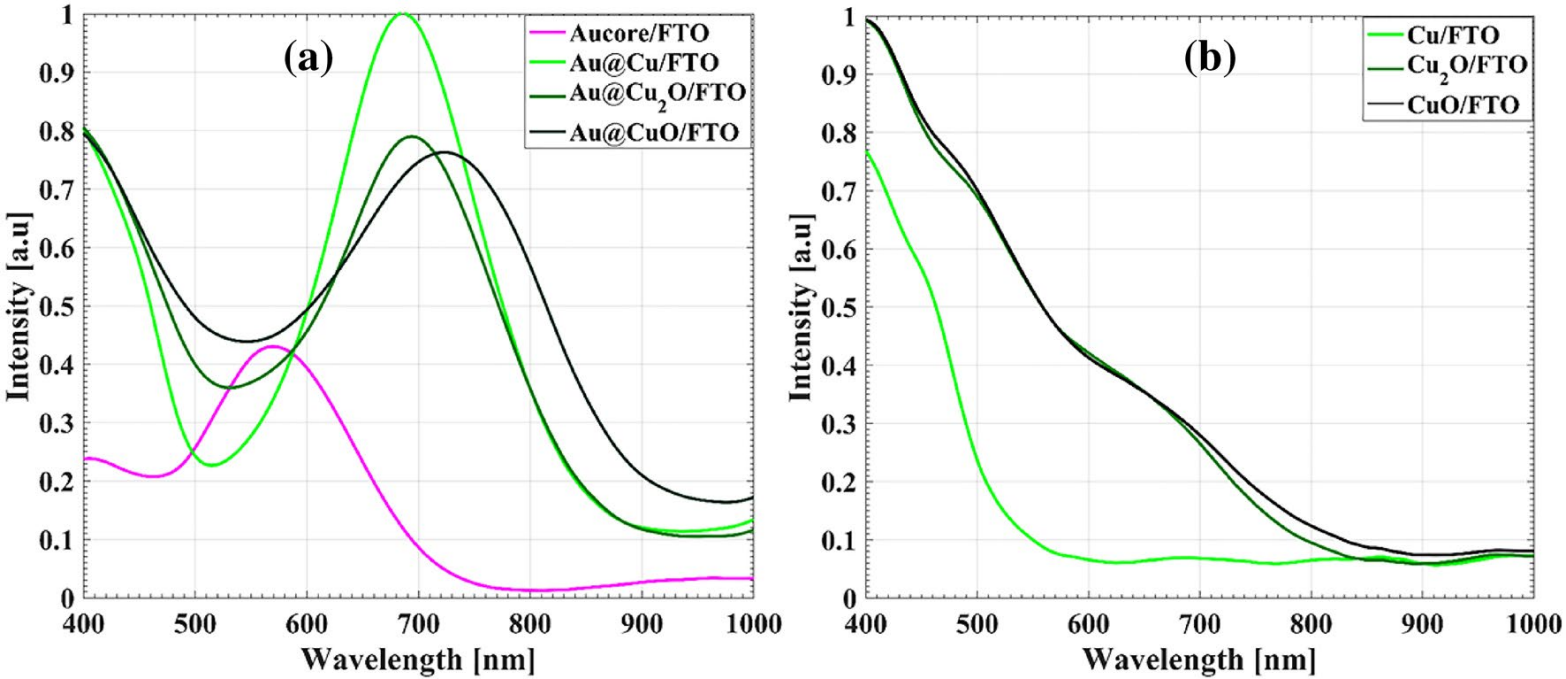
Vis–NIR spectrum of (**a**) Aucore supported FTO electrode, Au@cu, Au@cu_2_O and Au@CuO FTO electrodes and (**b**) Cu/FTO, Cu_2_O/FTO and CuO/FTO electrodes.

On the other hand, the formation of core–shell can also be investigated by Vis–NIR spectroscopy via changes of surface plasmon resonance (SPR) due to formation of Cu shell on Au core^47,48^. The Vis–NIR absorption spectra of Cu deposited on the bare and core supported FTO electrodes and the corresponding oxide forms are shown in Fig. 4. For Au nanocores, a plasmonic peak at the wavelength of 570 nm is observed. Deposition of Cu on the nanocores leads to two fold increment of the intensity, the redshift of the plasmonic peak to 683 nm and no splitting spectra indicating the formation of spherical Au@Cu^49^. The decrement of peak intensity and further redshift of plasmonic peak wavelength to 695 nm and 724 nm are observed by the formation of Au@Cu_2_O and Au@CuO, in agreement with previous reports^34,49–53^.

In the case of the Cu/FTO and their oxide states, no SPR peak is observed and only a broad absorption band in the range of 480–800 nm, associated with the bandgap of Cu_2_O and CuO, is recognized^34,53,54^.

The structure and composition characterizations of the Au@Cu core–shell and its oxide forms have been carried out by XRD, as shown in Fig. S4. XRD patterns could confirm the structure and composition of electrodes. XRD patterns of the bare and Au core supported FTO electrodes are also shown in the figure. The pattern of the FTO bare electrode shows several peaks at 2*θ* = 27.1°, 34.06°, 39°, 52.8°, 55.1°, 62°, 65.8°, 72° and 79.15°, which are assigned to the tetragonal structure of the FTO electrode. In the case of the Au nanocores, two diffraction peaks of 38.4° and 44.9° are attributed to the (111) and (200) planes of the face-centered cubic (FCC) structure of Au. For Au@Cu, the appearance of diffraction peaks at 2θ = 44°, 52.1° and 75° corresponds to (110), (111), and (200) planes indicating the formation of FCC crystalline Cu structure. For the Au@Cu_2_O, XRD peaks at 2θ = 37.3° and 43.2° of the (111) and (200) planes confirm the formation of Cu_2_O shell due to thermal treatment of Cu shell at the temperature of 200 °C. Also, diffraction peaks at 2θ = 32.4°, 36°, 38.5°, 49.7°, 58.7° are assigned to the (110), (− 111), (111), (− 202) and (202) planes of CuO at the temperature of 400 °C.

As a supplementary characterization, the Raman study was performed on the Au@Cu_x_O nanostructures where CuO and CuO phases are anticipated^45,55^. Figure S5 shows Raman spectrums of Au@Cu_2_O and Au@CuO nanostructures. For the Au@Cu_2_O case, five peaks have been observed at 213, 305, 420, 514, and 626 cm^−1^. The Raman spectrum of Au@CuO shows three peaks at 286, 342, and 620 cm^−1^. This result indicates that the Cu_2_O and CuO phases were acquired using the thermal processing agreeing well with the XRD results.

3. Electrocatalytic properties of Au@Cu and Au@CuxO towards glucose oxidation

**Figure 5:**
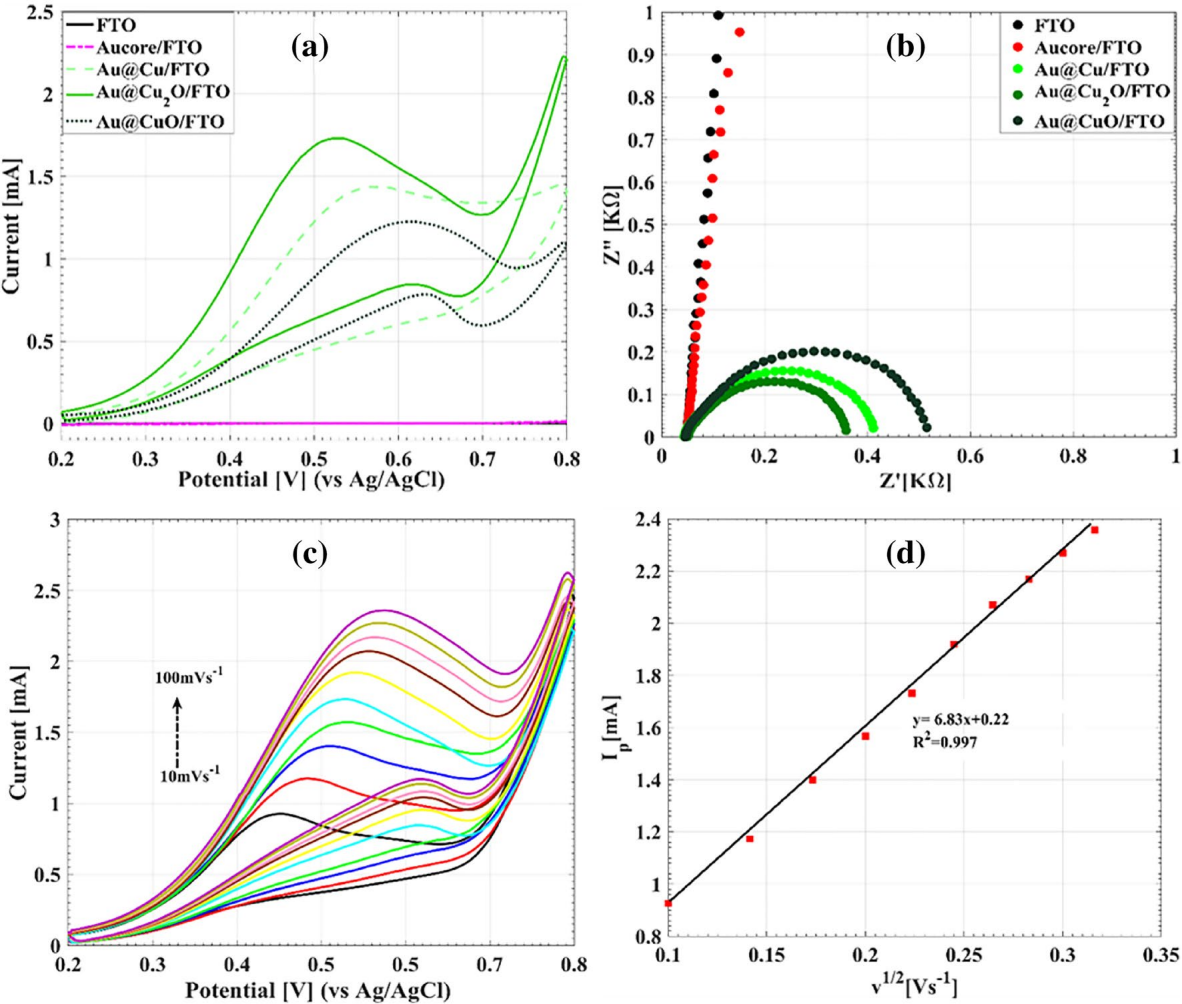
(**a**) The CV curves of different electrodes in present of 1 mM glucose in 0.1 M NaOH solution at scan rate of 50 mVs^−1^. (**b**) Nyquist plots of EIS spectra of different electrodes in the frequency range from 100 kHz to 0.1 Hz at the applied potential of 0.55 V. (**c**) The CV curves of the Au@Cu_2_O electrode at different scan rates in 0.1 M NaOH with 1 mM glucose. (**d**) Calibration curve of anodic peak currents versus the square root of the scan rate.

The electrocatalytic activity of the fabricated electrodes was studied by cyclic voltammetry in 0.1 M NaOH solution containing 1 mM glucose at the scan rate of 50 mVs^−1^, as shown in Fig. 5a. The bare and Au core supported FTO electrodes show no activity toward glucose oxidation in the studied potential range. A considerable glucose oxidation peak is followed for the Au@Cu and its oxidated forms. The precise mechanism for copper-based electrooxidation of glucose in the alkaline medium has not been thoroughly interpreted. Based on the latest proposed mechanism, the formation of a couple of adsorbed hydroxyl ions and the p-type CuO semiconductor cooperate in oxidation of glucose, which could be explained with the following reactions^56,57^:

1 $$\begin{gathered} OH^{ - }_{ads} + h^{ + } \to (OH^{ - }_{ads} )(h^{ + } ) \hfill \\ (OH^{ - }_{ads} )(h^{ + } ) + Glu\cos e \to H_{2} O + {\text{Gluconolactone}} \hfill \\ \end{gathered}$$

Although CuO species catalyze the oxidation process of glucose according to the above equation, the charge transfer ability is also a critical issue. As shown in Fig. 5a, Au@Cu_2_O/FTO shows higher reaction rates, considerably higher current, and a more negative peak potential shift than the other electrodes. The EIS studies were additionally used to compare the conductivity properties of electrodes. EIS measurements were performed in the frequency range from 100 kHz to 0.1 Hz at 0.55 V. As shown in the Nyquist plots of Fig. 5b, the smallest semicircle diameter and consequently the lowest charge-transfer resistance is obtained for Au@Cu_2_O. Regarding the results of Fig. 5a,b, the Au@Cu_2_O exhibits superior performance for glucose sensing applications. Consequently, this electrode was applied for more glucose sensing studies.

Figure 5c shows the effect of scan rate on the glucose oxidation efficiency of the Au@Cu_2_O electrode. The anodic current is considerably enhanced by increasing of the scan rate. The increment of the scan rate from 10 to 100 mVs^−1^ directs to the small shift of the peak potential from 0.45 to 0.57 V, indicating the rapid electron transfer of the electrode for glucose oxidation process. As indicated in Fig. 5d, the linear dependency of the peak current (I_p_) versus the square root of scan rate confirms a diffusion-controlled process due to the rapid electron transfer through the Au@Cu_2_O electrode.

### Optimization of sensor parameters

The applied potential and concentration of NaOH solution have notable impacts on the sensing performance of non-enzymatic glucose sensors. Therefore, to raise the glucose-sensing performance, the utilized potential was studied in a range from 0.2 to 0.6 V by measuring the amperometric current of the sensor under the subsequent addition of 0.5 mM glucose. As shown in Fig. S6a, amperometric current increases notably from the applied potential of 0.2 to 0.55 V and then decreases. Hence, the potential of + 0.55 V was selected as the optimized potential in the following experiments. Figure S6b shows the effect of various NaOH concentrations on the current response of the Au@Cu_2_O electrode under the subsequent addition of glucose at the potential of + 0.55 V. As can be seen, the amperometric currents are the same for all NaOH concentrations after the first addition of glucose. The difference becomes more distinguishable after the addition of more glucose. Finally, the highest current response is obtained for 0.1 M NaOH concentration.

**Figure 6:**
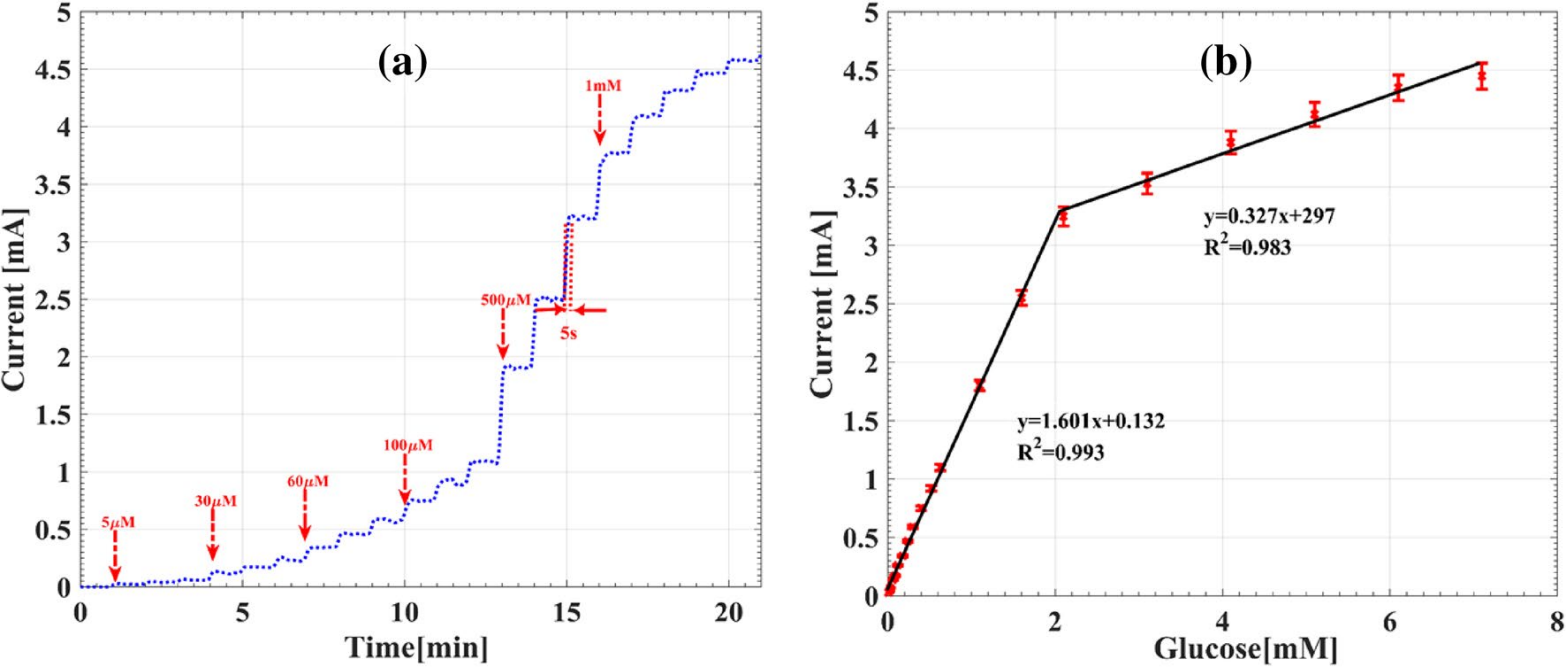
(**a**) Amprometric response of the Au@Cu_2_O electrode after successive addition of glucose into 0.1 M NaOH. (**b**) The current response versus glucose concentration for this electrode.

To investigate the sensing properties, the amperometric response of the Au@Cu_2_O electrode was recorded at potential of + 0.55 V in 0.1 M NaOH solution under the subsequent addition of glucose with different concentrations, as shown in Fig. 6a. A fast response time of 5 s is observed for reaching the steady state current value. The corresponding calibration curve of Fig. 6b illustrates two linear ranges from 5 μM to 2.1 mM and from 2.1 to 7 mM. For the first linear range, the sensitivity and the limit of detection (LOD) are acquired as 1601 μAcm^−2^ mM^−1^ and 0.6 μM, respectively. The equation of 3σ_b_/s was used to calculate the detection limit, where σ_b_ is the standard deviation of the blank solution and s is the slope of the calibration curve line, respectively. A sensitivity of 327 μAcm^−2^ mM^−1^ is determined for the linear range of 2.1 mM–7 mM. Table 1 shows the comparison of the sensing properties of our proposed Au@Cu_2_O electrode with some recently reported non-enzymatic glucose sensors. Considering the results of Table 1, the superiority of the sensing performance of the Au@Cu_2_O electrode is confirmed, specifically as regards sensitivity, LOD, and response time. This remarkable sensing performance is originated from three reasons: (1) excellent charge transfer due to direct deposition of core on the surface of the bare electrode compared to other conventional core–shell stabilization methods, (2) higher reaction capacity of shell layer as a result of direct deposition and selective growth and (3) existence of more accessible active sites for electrochemical reactions.

**Table 1:** Comparison of electrochemical performance of recently reported non-enzymatic glucose sensor.

Electrode	Potential (V)	Reference electrode	Linear range (mM)	Sensitivity μAcm^−2^ mM^−1^	LOD (μM)	Response time	References
Au@Cu_2_O/Nafion/GCE	0.5–0.6	Ag/AgCl	0.05–2	715	18	–	^34^
Cu-Cu_2_O/GCE	0.55	SCE	0.002–4	1446	1.4	$\sim$ 1.3	^58^
Cu_2_O-RD/GCE	0.6	Ag/AgCl	0.1–8.4	1104.3	0.639	–	^59^
Barnyardgrass-like CuO/Cu_2_O	0.4–0.5	Ag/AgCl	0.05–2	1281	16.7	–	^60^
Cuboctahedral Cu_2_O/GCE	0.5–0.6	Ag/AgCl	0.005–12	309	3.5	–	^61^
3D Cu@Cu_2_O/GCE	0.6	Ag/AgCl	0.001–17.12	194.88	54	–	^62^
Cu_2_O/RGO	0.6	Ag/AgCl	0.1–9	813.713	0.44	–	^63^
Cu@Cu_2_O/GCE	0.55	Ag/AgCl	0.05–8	–	15	–	^64^
AuNPs@CuONWs/Cu_2_O/CF	0.21	Ag/AgCl	0.0028–2	1619	0.9	< 5	^65^
Au@Cu_2_O/FTO	0.55	Ag/AgCl	0.005–2.1 2.1–7	1601 327	0.6	~ 5	This work

GCE: glassy carbon electrode; CPE: carbon paste electrode; RD: rhombic dodecahedral; RGO: reduced graphene oxide; NPs: Au nanoparticles; NWs: nanowires; CF: copper foam.

### Selectivity, reproducibility and stability tests

A major challenge of non-enzymatic glucose sensing devices is their anti-interference ability which affects the sensing performance in real sample monitoring. In order to verify the selectivity of the Au@Cu_2_O electrode, the sensor response was measured in the presence of some electroactive species in 0.1 M NaOH solution at the potential of + 0.55 V. The amperometric response was recorded under the addition of 0.5 mM glucose and interfering species including 0.02 mM UA, 0.1 mM AA, and 0.1 mM lactose, 0.1 mM sucrose, 0.1 mM mannose, 0.1 mM fructose, as shown in Fig. S7a. The results show a negligible current response for interfering species in comparison to that of glucose. These results suggest affordable performance of the Au@Cu_2_O electrode for the detection of glucose in real samples.

Reproducibility and stability are other vital parameters for assessing the efficiency of a sensor. The reproducibility of five prepared electrodes was investigated by recording the current response for 1 mM glucose in 0.1 M NaOH solution. The relative standard deviation (RSD) of 3.8% for five prepared electrodes indicates an excellent reproducibility of the sensor owning to purposed processes for fabrication of the core–shell structure. In addition, the stability of the sensor was also investigated by monitoring of current response for one month. As shown in Fig. S7b, the sensor indicates good stability with remaining about 92% of its initial current response after one month.

### Practical utility of the sensor in real sample analysis

As a real application test, three serum samples with known glucose concentrations of 4.8, 4.26, and 5.63 mM were evaluated by the Au@Cu_2_O sensors (one electrode per sample). The chronoamperometry method at the voltage of + 0.55 V was employed for 20 mL NaOH solution (0.1 M, pH = 13) under stirring. A 200 μL of each serum sample was separately added to the 20 mL NaOH solution for each sample test and then the current response was recorded. Finally, the glucose concentration was calculated utilizing the equation of y = 1.601 x + 0.132 (which was obtained from the first linear range of the sensor calibration curve) with x and y as the glucose concentration (mM) and the measured current (mA), respectively. The results were compared with the referenced values obtained by a biochemical analysis method, as showed in Table S1. The obtained results are in fine agreement with the data from the biochemical analysis with less than 3.4% deviation. Moreover, the recovery test results show the proper performance of the fabricated electrode. Accordingly, this new proposed process for the fabrication of core–shell structure has excellent potential for the assay of glucose in real samples.

## Conclusions

In summary, we have developed a novel approach that produces Au@Cu and Au@Cu_x_O nanostructures directly on FTO electrodes. The Au core nanoparticles were synthesized by the physical vapor deposition followed with a thermal treatment. Also, Cu shell structures are easily deposited and grown on the Au cores by electrochemical deposition. The developed electrode has unique properties including fast, highly controllable and precise fabrication scheme. Surface plasmon were observed from these nanostructures, which confirm constituting core–shell structure. Additionally, Au@Cu_2_O and Au@CuO have been controllably fabricated via post thermal annealing of Au@Cu. Electrochemical properties of different core–shell structures toward glucose oxidation have been investigated. Based on the electrochemical measurements, Au@Cu_2_O indicates the highest electrocatalytic activity regarding oxidation of glucose. The Au@Cu_2_O electrode depicts a high sensitivity of 1601 μAcm^−2^ mM^−1^, a low detection limit of 0.6 μM and two linear response ranges between 5 μM–2.1 mM and 2.1–7 mM. Also, the sensor indicates remarkable reproducibility and supplies accurate results for glucose detection in human serums. The results propose useful new ideas for developing core–shell structures with different morphology and sizes by adjusting electrochemical deposition and thermal treatment parameters.

## Methods

### Chemicals and materials

Au metallic pellets (99.99% purity) that have been used in this study were bought from Kurt J. Lesker. Fluorine-doped tin oxide (FTO) sheets with 2.2 mm thickness and 15 Ωsq^−1^ surface resistance were obtained from Nanogostar Sepahan and used as the supporting electrode. Selection of FTO as the substrate instead of using common glassy carbon and screen-printed electrodes, is based on a number of advantages of FTO including cost, stability against thermal treatment, and optical transparency, which is required in optical characterization of Au@Cu_x_O nanostructures. Copper sulfate (CuSO_4_), potassium ferricyanide [K_3_Fe (CN)_6_], potassium ferrocyanide [K_4_Fe (CN)_6_], potassium chloride (KCl), sodium hydroxide (NaOH), glucose, and other interference species were acquired from Sigma-Aldrich. Milli-Q water (18.2 MΩcm) was employed throughout this work to prepare all solutions.

### Instruments

All electrochemical experiments were followed through an Origalys (ElectroChem SAS, France) potentiostat/galvanostat system. The electrochemical measurements were executed in a conventional three-electrode cell with a modified working electrode, a platinum foil counter electrode and an Ag/AgCl reference electrode. The surface morphologies were described by a field emission scanning electron microscopy (FESEM), MIRA3 TESCAN-XMU. Composition evolutions of nanostructures were characterized using grazing incidence X-ray diffraction (XRD) with Cu- Kα radiation. The absorption spectra were recorded by Unico 4802 UV–Vis-NIR spectrophotometer.

### Fabrication of Au cores on the FTO substrate

To produce Au cores, Au thin film was evaporated on the FTO substrate. The physical vapor deposition parameters were according to a procedure described previously^43^. After that, the Au cores were formed on the FTO substrate by annealing the Au film at 550 °C for 10 h under the air atmosphere^43^. To study the influence of Au film thickness on the charge transfer properties of the fabricated electrode, various thicknesses of 2.5, 5 and 10 nm have been deposited.

### Fabrication of Au@Cu and Au@Cu_X_O structures

To produce Au@Cu and Au@Cu_x_O, a copper shell was electrochemically deposited on the Au core supported FTO substrate followed by thermal annealing at various temperatures. The electrochemical deposition of the copper shell was performed under potentiostatic mode in 1 mM CuSO_4_ solution at room temperature. The deposition potential and deposition time were fixed at − 0.45 V and 600 s, respectively. Finally, to obtain Au@Cu_2_O and Au@CuO, the as-prepared Au@Cu were annealed under the air atmosphere at 200 °C and 400 °C, respectively.

### Human serum samples preparation for glucose tests

Blood serums were acquired from three individual males using the following method. Each blood sample was collected in an individual serum separator tube and allowed to clot for 30 min at room temperature. Afterward, the samples were centrifuged at 3000 rpm for 15 min to remove the clot. Ultimately, the resulting serums were stored at − 20 °C in refrigerator.

### Ethical approval

All sample collection protocols were conducted according to the ethical principles of Tarbiat Modares University and the study was approved by the ethics committee of Tarbiat Modares University. All experiments and methods were executed under the relevant guidelines and regulations authorized by the ethics committee of Tarbiat Modares University. A written informed consent was obtained from the participants in the blood glucose tests.

## Supplementary Information

Supplementary Information.

## Data Availability

All data reported during this study are contained in this published article and its supplementary information file.

## References

[1] Heller A, Feldman B (2008). Electrochemical glucose sensors and their applications in diabetes management. Chem. Rev..

[2] Chen C (2013). Recent advances in electrochemical glucose biosensors: A review. RSC Adv..

[3] Fragkou V (2010). Improvements in electrochemical glucose biosensors. Chem. Rev..

[4] Cinti S, Marrone R, Mazzaracchio V, Moscone D, Arduini F (2020). Novel bio-lab-on-a-tip for electrochemical glucose sensing in commercial beverages. Biosens. Bioelectron..

[5] Shi L, Zhu X, Liu T, Zhao H, Lan M (2016). Encapsulating Cu nanoparticles into metal-organic frameworks for nonenzymatic glucose sensing. Sens. Actuators B Chem..

[6] Wang J (2001). Glucose biosensors: 40 years of advances and challenges. Electroanal. Int. J. Devoted Fundam. Pract. Asp. Electroanal..

[7] Nery EW, Kundys M, Jeleń PS, Jönsson-Niedziólka M (2016). Electrochemical glucose sensing: Is there still room for improvement?. Anal. Chem..

[8] Muguruma H, Hoshino T, Nowaki K (2015). Electronically type-sorted carbon nanotube-based electrochemical biosensors with glucose oxidase and dehydrogenase. ACS Appl. Mater. Interfaces.

[9] Oliver NS, Toumazou C, Cass AEG, Johnston DG (2009). Glucose sensors: A review of current and emerging technology. Diabet. Med..

[10] Ahmad R (2017). Highly efficient non-enzymatic glucose sensor based on CuO modified vertically-grown ZnO nanorods on electrode. Sci. Rep..

[11] Lee WC (2019). Comparison of enzymatic and non-enzymatic glucose sensors based on hierarchical Au-Ni alloy with conductive polymer. Biosens. Bioelectron..

[12] Sajadpour M (2019). A non-enzymatic glucose sensor based on the hybrid thin films of Cu on acetanilide/ITO. J. Electrochem. Soc..

[13] Hwang DW, Lee S, Seo M, Chung TD (2018). Recent advances in electrochemical non-enzymatic glucose sensors – A review. Anal. Chim. Acta.

[14] Wei M (2020). Electrochemical non-enzymatic glucose sensors: Recent progress and perspectives. Chem. Commun..

[15] Wang G (2013). Non-enzymatic electrochemical sensing of glucose. Microchim. Acta.

[16] Tee SY, Teng CP, Ye E (2017). Metal nanostructures for non-enzymatic glucose sensing. Mater. Sci. Eng. C.

[17] Zaidi SA, Shin JH (2016). Recent developments in nanostructure based electrochemical glucose sensors. Talanta.

[18] Pak, M., Moshaii, A., Siampour, H., Abbasian, S. & Nikkhah, M. Cobalt-copper bimetallic nanostructures prepared by glancing angle deposition for non-enzymatic voltammetric determination of glucose. *Microchim. Acta* **187**, (2020). 10.1007/s00604-020-04246-2 32307592

[19] Dong Q, Ryu H, Lei Y (2021). Metal oxide based non-enzymatic electrochemical sensors for glucose detection. Electrochim. Acta.

[20] Lv J (2017). Facile synthesis of novel CuO/Cu2O nanosheets on copper foil for high sensitive nonenzymatic glucose biosensor. Sens. Actuators B Chem..

[21] Cheng S (2019). Hierarchical Co3O4/CuO nanorod array supported on carbon cloth for highly sensitive non-enzymatic glucose biosensing. Sens. Actuators B Chem..

[22] Wang X (2010). Synthesis of CuO nanostructures and their application for nonenzymatic glucose sensing. Sens. Actuators B Chem..

[23] Jayasingha L (2020). Nanoporous Cu2O nanotube/nanorod array electrodes for non-enzymatic glucose sensing with high sensitivity and very low detection limit. Electrochim. Acta.

[24] Singer N, Pillai RG, Johnson AID, Harris KD, Jemere AB (2020). Nanostructured nickel oxide electrodes for non-enzymatic electrochemical glucose sensing. Microchim. Acta.

[25] Xu J (2019). Co 3 O 4 nanostructures on flexible carbon cloth for crystal plane effect of nonenzymatic electrocatalysis for glucose. Biosens. Bioelectron..

[26] Dayakar T (2018). Novel synthesis and characterization of Ag@TiO 2 core shell nanostructure for non-enzymatic glucose sensor. Appl. Surf. Sci..

[27] Mishra AK (2020). Au nanoparticles modified CuO nanowire electrode based non-enzymatic glucose detection with improved linearity. Sci. Rep..

[28] Khairullina, E. *et al.* Laser-assisted surface activation for the fabrication of flexible non-enzymatic Cu-based sensors. (2022). 10.1007/s00604-022-05347-w 35704127

[29] Wang S (2021). Flowerlike CuO/Au nanoparticle heterostructures for nonenzymatic glucose detection. ACS Appl. Nano Mater..

[30] Chakraborty P, Dhar S, Debnath K, Majumder T, Mondal SP (2019). Non-enzymatic and non-invasive glucose detection using Au nanoparticle decorated CuO nanorods. Sens. Actuators B Chem..

[31] Muench F (2017). Free-standing networks of core-shell metal and metal oxide nanotubes for glucose sensing. ACS Appl. Mater. Interfaces.

[32] Hsu CL, Fang YJ, Hsueh TJ, Wang SH, Chang SJ (2017). Nonenzymatic glucose sensor based on Au/ZnO core-shell nanostructures decorated with Au nanoparticles and enhanced with blue and green light. J. Phys. Chem. B.

[33] Li Z, Xin Y, Zhang Z, Wu H, Wang P (2015). Rational design of binder-free noble metal/metal oxide arrays with nanocauliflower structure for wide linear range nonenzymatic glucose detection. Sci. Rep..

[34] Su Y (2018). Au@Cu2O core-shell structure for high sensitive non-enzymatic glucose sensor. Sens. Actuators B Chem..

[35] Sehit E, Altintas Z (2020). Significance of nanomaterials in electrochemical glucose sensors: An updated review (2016–2020). Biosens. Bioelectron..

[36] Messaoudi O (2015). Synthesis and characterization of ZnO/Cu2O core–shell nanowires grown by two-step electrodeposition method. Appl. Surf. Sci..

[37] Zhang P (2018). Dendritic core-shell nickel-iron-copper metal/metal oxide electrode for efficient electrocatalytic water oxidation. Nat. Commun..

[38] Wei H (2021). Dendritic core-shell copper-nickel alloy@ metal oxide for efficient non-enzymatic glucose detection. Sens. Actuators B Chem..

[39] Fan F-R (2008). Epitaxial growth of heterogeneous metal nanocrystals: Ffrom gold nano-octahedra to palladium and silver nanocubes. J. Am. Chem. Soc..

[40] Tsuji M, Yamaguchi D, Matsunaga M, Alam MJ (2010). Epitaxial growth of Au@Cu core-shell nanocrystals prepared using the PVP-assisted polyol reduction method. Cryst. Growth Des..

[41] Xia Y, Gilroy KD, Peng H, Xia X (2017). Seed-mediated growth of colloidal metal nanocrystals. Angew. Chemie Int. Ed..

[42] Qin Y (2008). Ionic liquid-assisted growth of single-crystalline dendritic gold nanostructures with a three-fold symmetry. Chem. Mater..

[43] Siampour H (2020). Seed-mediated electrochemically developed Au nanostructures with boosted sensing properties: An implication for non-enzymatic glucose detection. Sci. Rep..

[44] Wang T, Su W, Fu Y, Hu J (2016). Controllably annealed CuO-nanoparticle modified ITO electrodes: Characterisation and electrochemical studies. Appl. Surf. Sci..

[45] Figueira J (2017). Optimization of cuprous oxides thin films to be used as thermoelectric touch detectors. ACS Appl. Mater. Interfaces.

[46] Grujicic D, Pesic B (2002). Electrodeposition of copper: The nucleation mechanisms. Electrochim. Acta.

[47] Alvarez-Paneque AF, Rodríguez-González B, Pastoriza-Santos I, Liz-Marzán LM (2013). Shape-templated growth of Au@ Cu nanoparticles. J. Phys. Chem. C.

[48] Monzó J (2015). Enhanced electrocatalytic activity of Au@ Cu core@ shell nanoparticles towards CO 2 reduction. J. Mater. Chem. A.

[49] Liu DY (2012). Distinctive enhanced and tunable plasmon resonant absorption from controllable Au@Cu 2O nanoparticles: Experimental and theoretical modeling. J. Phys. Chem. C.

[50] Zhang L, Jing H, Boisvert G, He JZ, Wang H (2012). Geometry control and optical tunability of metal-cuprous oxide core-shell nanoparticles. ACS Nano.

[51] Kumar DR, Manoj D, Santhanalakshmi J, Shim JJ (2015). Au-CuO core-shell nanoparticles design and development for the selective determination of Vitamin B6. Electrochim. Acta.

[52] Zhao Y (2018). Shell-encoded Au nanoparticles with tunable electroactivity for specific dual disease biomarkers detection. Biosens. Bioelectron..

[53] Kuo MY (2019). Au@Cu2O core@shell nanocrystals as dual-functional catalysts for sustainable environmental applications. Appl. Catal. B Environ..

[54] Mousavi-Kamazani M, Zarghami Z, Rahmatolahzadeh R, Ramezani M (2017). Solvent-free synthesis of Cu-Cu2O nanocomposites via green thermal decomposition route using novel precursor and investigation of its photocatalytic activity. Adv. Powder Technol..

[55] Zoolfakar AS, Rani RA, Morfa AJ, O’Mullane AP, Kalantar-Zadeh K (2014). Nanostructured copper oxide semiconductors: A perspective on materials, synthesis methods and applications. J. Mater. Chem. C.

[56] Barragan JTC, Kogikoski S, Da Silva ETSG, Kubota LT (2018). Insight into the electro-oxidation mechanism of glucose and other carbohydrates by CuO-based electrodes. Anal. Chem..

[57] Siampour H, Abbasian S, Moshaii A (2020). Copper columnar nanostructures fabricated by glancing angle deposition as a robust and scalable method for high sensitive non-enzymatic glucose detection. Appl. Surf. Sci..

[58] Cao K (2020). Boosting glucose oxidation by constructing Cu-Cu2O heterostructures. New J. Chem..

[59] Hong BD, Lee CL (2020). Specific activities of rhombic dodecahedral, octahedral, and cubic Cu2O nanocrystals as glucose oxidation catalysts. Chem. Eng. J..

[60] Zhou QQ (2019). Controllable synthesis of barnyardgrass-like CuO/Cu2O heterostructure nanowires for highly sensitive non-enzymatic glucose sensors. J. Mater. Chem. C.

[61] Jiang Y (2021). Facet-dependent Cu2O electrocatalysis for wearable enzyme-free smart sensing. ACS Catal..

[62] Gao Y (2019). Three-dimensional porous Cu@Cu 2 O aerogels for direct voltammetric sensing of glucose. Microchim. Acta.

[63] Wang Y (2017). Facile growth of Cu2O hollow cubes on reduced graphene oxide with remarkable electrocatalytic performance for non-enzymatic glucose detection. New J. Chem..

[64] Ling P, Zhang Q, Cao T, Gao F (2018). versatile three-dimensional porous Cu@Cu 2 O aerogel networks as electrocatalysts and mimicking peroxidases. Angew. Chemie.

[65] Zhao Z (2021). Highly sensitive and portable electrochemical detection system based on AuNPs@CuO NWs/Cu2O/CF hierarchical nanostructures for enzymeless glucose sensing. Sens. Actuators B Chem..

