# Supplementary material for: Stable, reproducible, and binder-free gold/copper core–shell nanostructures for high-sensitive non-enzymatic glucose detection
Source: Sci Rep. 2022 Nov 8;12:18945. doi: 10.1038/s41598-022-23504-2 (PMC9643390; doi:10.1038/s41598-022-23504-2)
Supplement: Supplementary file 1 — Supplementary Information. [file 41598_2022_23504_MOESM1_ESM.docx]

Supplementary Information

**Stable, reproducible, and binder-free gold/copper core-shell nanostructures for high-sensitive non-enzymatic glucose detection**

Hossein Siampour^1^, Sara Abbasian^2^, Ahmad Moshaii^1, 2*^, Amir R. Amirsoleimani^1^

^1^Department of Physics, Tarbiat Modares University, Tehran, P.O Box 14115-175, Iran

^2^Department of Sensor and Biosensor, Faculty of Interdisciplinary Sciences and Technologies, Tarbiat Modares University, P.O. Box: 14115-336, Tehran, Iran

* E-mail: [moshaii@modares.ac.ir](mailto:moshaii@modares.ac.ir)


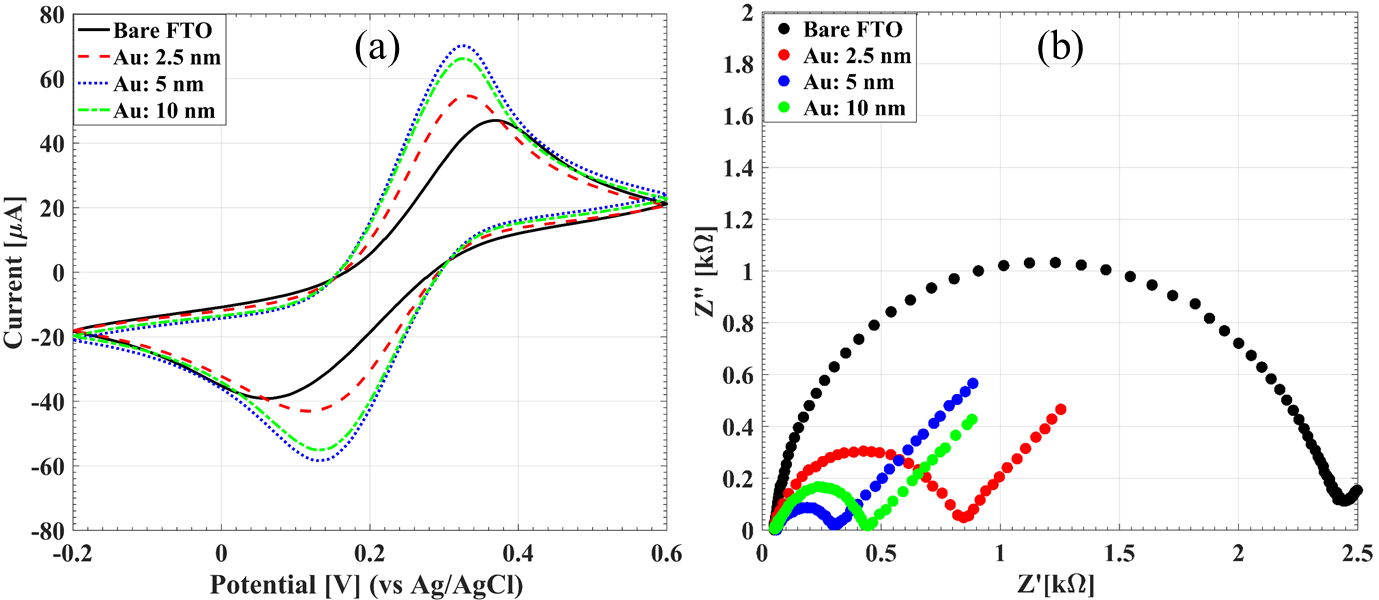


**Figure S1**. Cyclic voltammograms (a) and Nyquist plots (b) of the bare FTO electrode and the Au nanocores electrodes with deposition thicknesses of 2.5, 5 and 10 nm in 0.1 M KCl solution containing redox couple of [Fe (CN)_6_] ^-3/-4^.


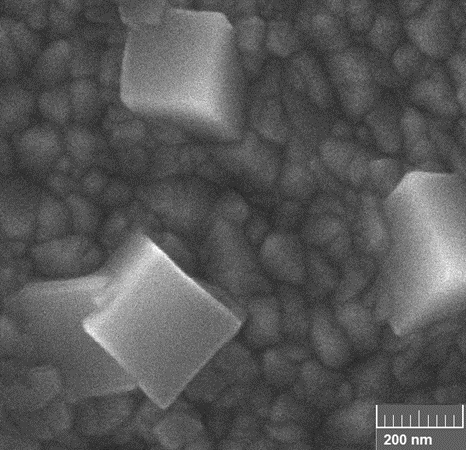


**Figure S2**. SEM image of the copper deposited on the FTO electrode at the potential -0.45 V for 600 s in 1 mM CuSO_4_ electrolyte solution.


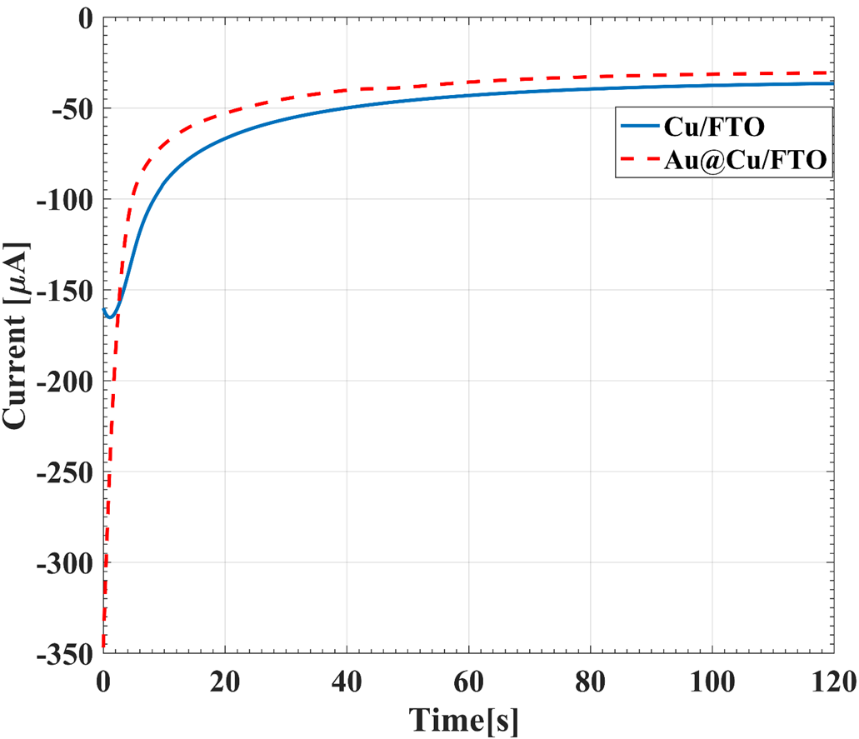


**Figure S3**. The current transient curves of Cu deposition on the bare and the Au core supported FTO electrodes.


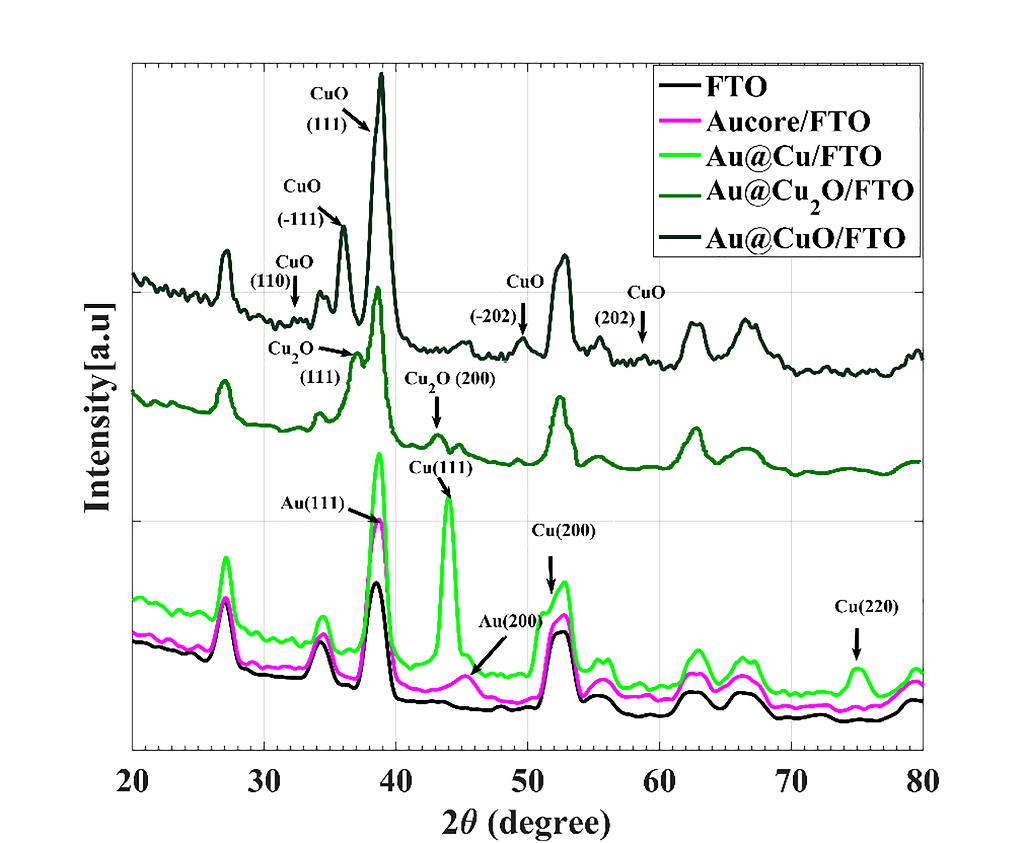


Figure S4. XRD pattern of the bare FTO electrode, Au core supported FTO electrode, Au@Cu, Au@Cu_2_O and Au@CuO FTO electrodes.


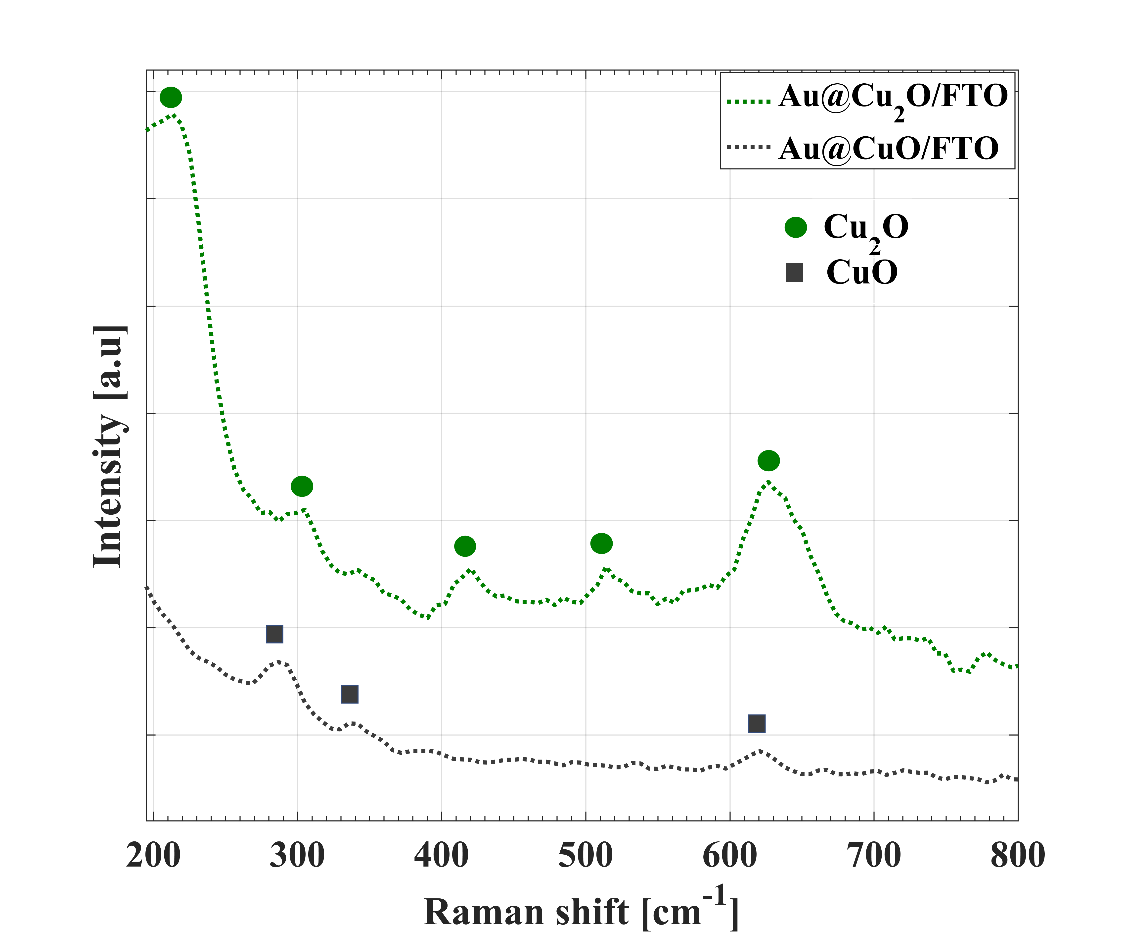


**Figure S5**. Raman spectra of the Au@Cu_2_O, and the Au@CuO structures on the FTO electrodes.


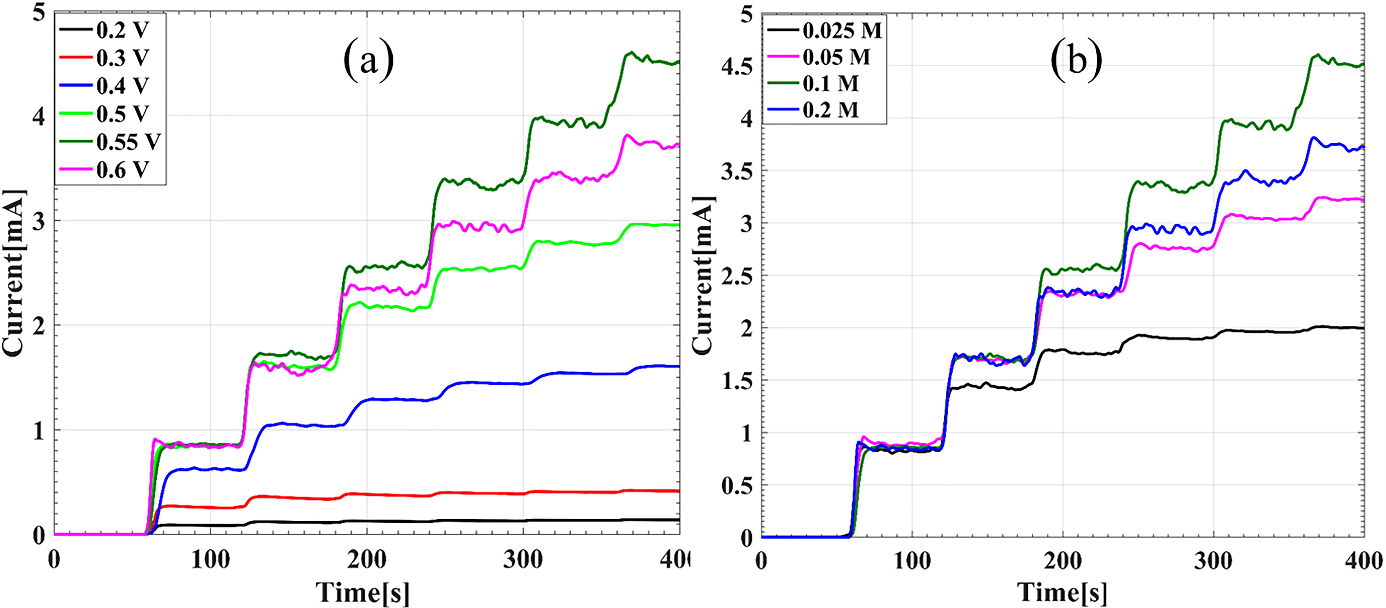


Figure S6. Amperometric response of the Au@Cu_2_O electrode after successive addition of 0.5 mM glucose at different applied potentials in 0.1 M NaOH solution (a), and different concentrations of NaOH solution at the applied potential of +0.55 V (b).


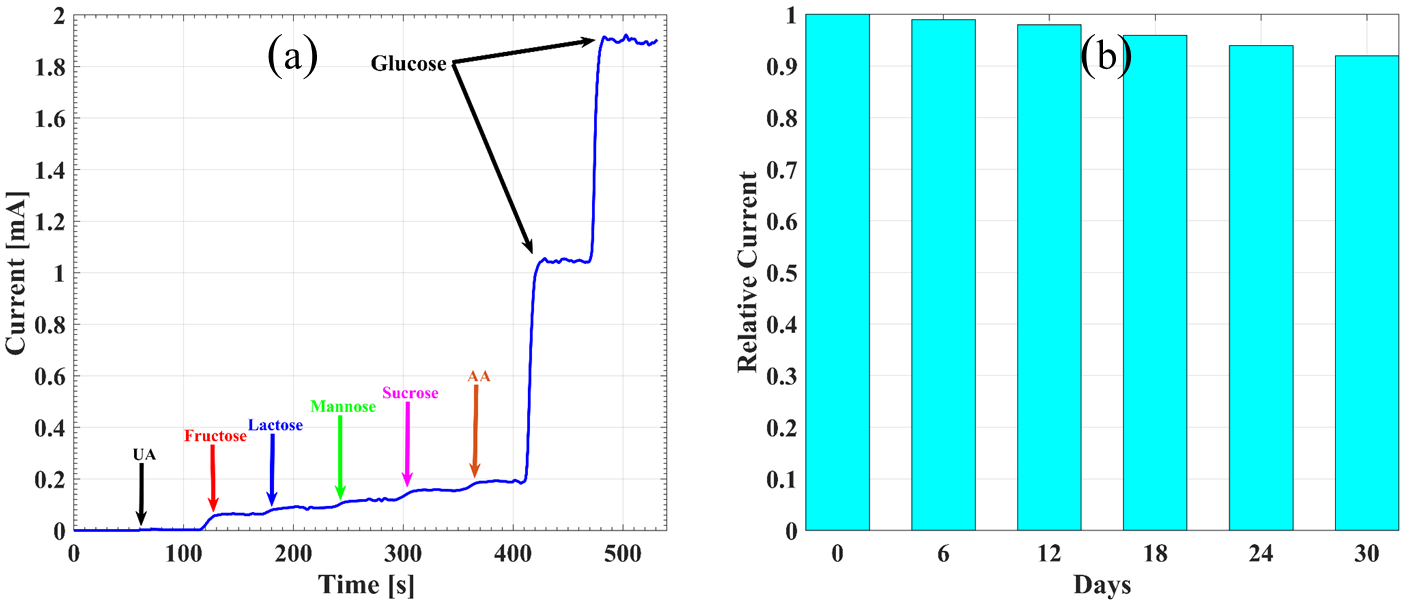


**Figure S7**. (a) Selectivity of Au@Cu_2_O electrode in present of interference species including 0.02 mM UA, 0.1 mM fructose, 0.1 mM lactose, 0.1 mM mannose, 0.1 mM sucrose, 0.1 mM AA and addition of 0.5 mM glucose at applied potential of + 0.55 V and (b) stability Of Au@Cu_2_O electrode in present of 1mM glucose during one month.

**Table S1.** Detection results of glucose in human serum samples

| Samples | Clinical results (mM) | This sensor  (mM) | Difference (%) | RSD (n = 3)  (%) | Glucose added  (mM) | Recovery test  (%) |
| --- | --- | --- | --- | --- | --- | --- |
| Serum 1 | 4.80 | 4.65 | 3.12 | 2.84 | 0 |  |
|  |  |  |  |  | 0.5 | 99 |
|  |  |  |  |  | 1 | 104 |
| Serum 2 | 4.26 | 4.12 | 3.28 | 3.05 | 0 |  |
|  |  |  |  |  | 0.5 | 101 |
|  |  |  |  |  | 1 | 105 |
| Serum 3 | 5.63 | 5.44 | 3.37 | 3. 43 | 0 |  |
|  |  |  |  |  | 0.5 | 101 |
|  |  |  |  |  | 1 | 104 |
